# Supplementary material for: Genetic Diversity of Juglans mandshurica Populations in Northeast China Based on SSR Markers
Source: Front Plant Sci. 2022 Jun 30;13:931578. doi: 10.3389/fpls.2022.931578 (PMC9280368; doi:10.3389/fpls.2022.931578)
Supplement: Supplementary file 1 [file Table_1.DOCX]

Table S1 Information of 240 synthesized primers in *J. mandshurica*

| Number | ID | SSR | Forward primer (5'-3') | Tm (℃） | Reverse primer (5'-3') | Tm (℃） | Product size (bp) |
| --- | --- | --- | --- | --- | --- | --- | --- |
| 1 | TRINITY_DN1008_c1_g1_i69 | (CT)10 | TGTTGCCTCTTGCCACTATG | 59.86 | TTCATCCTTGACTCGCAGTG | 59.98 | 195 |
| 2 | TRINITY_DN101009_c0_g1_i1 | (TC)14 | CCCAGGGGATTCTCAATGTA | 59.74 | AAGTTTCCCCTCAAGAAAACG | 59.62 | 268 |
| 3 | TRINITY_DN1013_c1_g1_i1 | (CT)11 | TGGACTCTTTCTTTGCACCA | 59.42 | ATCAACAGACAACCCCAACC | 59.68 | 266 |
| 4 | TRINITY_DN1020_c0_g1_i8 | (TCG)5 | AGAAACGGTGTCGTTCTCGT | 59.77 | CCTCAGGCTCACGGAGATAG | 59.97 | 275 |
| 5 | TRINITY_DN1021_c0_g2_i1 | (AG)10 | TGAAAAGCCCTAATGAGGAAG | 58.45 | ATGCTGGCCTGATGGTTATC | 59.92 | 254 |
| 6 | TRINITY_DN102355_c0_g3_i2 | (TA)6 | AAGGAAGCTTTCAGCACAGC | 59.76 | CAATGAGCCCACTGATCAAA | 59.65 | 189 |
| 7 | TRINITY_DN1032_c0_g1_i20 | (AG)7 | AAACACGCAAGAGAGAGGGA | 59.99 | AGCAGCGTGAGAGAGAGGAC | 59.89 | 264 |
| 8 | TRINITY_DN1034_c0_g1_i47 | (GA)11 | TACGGTTTGGGGATAGCTTG | 59.95 | ACGAACAGTAACGGGGTCAC | 59.89 | 194 |
| 9 | TRINITY_DN1057_c0_g2_i12 | (AT)12 | TGTTGCTCATGCCTTGACTC | 59.99 | TTGTTAAGGTTGGAGAACTTAGGG | 59.93 | 268 |
| 10 | TRINITY_DN1058_c0_g1_i18 | (TC)7 | CTCGTTGCACCACCTCTGTA | 59.90 | CAGAGACACGGAGAAAGAAGG | 59.07 | 249 |
| 11 | TRINITY_DN10618_c0_g3_i1 | (AT)10 | TCCACCTAACTAGCGAAAGGA | 58.98 | ATGGCAGTTTGGTAGGCATC | 59.96 | 247 |
| 12 | TRINITY_DN1070_c0_g1_i10 | (GA)9 | GGTGTCATAGCCTCCTCTGC | 59.83 | ATGATGGGCCACAACTTAGC | 59.96 | 266 |
| 13 | TRINITY_DN107108_c3_g1_i1 | (AG)7 | TGACTCCCACTGTCTTGTGAG | 58.87 | ACTCATCATCCCACACACCA | 59.80 | 261 |
| 14 | TRINITY_DN1075_c0_g2_i1 | (GA)6 | GGAACTTCAGCTTGGTCGAG | 59.99 | GAGAAAGAAGCCAAGCAAACA | 59.62 | 228 |
| 15 | TRINITY_DN1077_c0_g2_i2 | (AG)12 | AAACTGGTATGGAGGATGCG | 59.96 | AGGGCTTCTTGGTTTCAATG | 59.17 | 245 |
| 16 | TRINITY_DN1088_c0_g2_i1 | (GA)6 | ATCACCACGAAGTCCGAATC | 59.93 | GAGGACGAAAACGTTACCCA | 59.97 | 200 |
| 17 | TRINITY_DN109208_c1_g1_i1 | (TA)8 | GGGACCAAACTCCTTGAACA | 59.94 | CTGGAAGATTTGCACGAACA | 59.84 | 263 |
| 18 | TRINITY_DN1096_c5_g1_i1 | (AG)10 | TCACGGTCCACCAATAAACA | 59.82 | CAGTCAGCGCATAAGGCATA | 60.00 | 255 |
| 19 | TRINITY_DN112373_c0_g1_i1 | (AG)6 | AAAGGAAAGCTGTTGGAGCA | 59.99 | TCGGTGTTTCGTCTTCTCTTC | 59.46 | 196 |
| 20 | TRINITY_DN112620_c0_g1_i1 | (TA)8 | ATTTCTGGACGCCAACATTC | 59.94 | GTGGGCTTTCTTTGTCTTCG | 59.85 | 201 |
| 21 | TRINITY_DN112976_c0_g1_i1 | (CGG)8 | ACTTTGTCGACCGATGGAAC | 59.97 | TGGTCAACTCTGGAGAGCCT | 59.99 | 274 |
| 22 | TRINITY_DN11415_c0_g1_i4 | (AAT)5 | CCTTTTGATACGAGAATCGG | 57.31 | TCAGAAATGGGAGAAATGGG | 59.86 | 229 |
| 23 | TRINITY_DN11455_c0_g1_i9 | (CT)6 | CCTGACCGTCTGGTAGAACC | 59.57 | CCACCAGCTGGAAAGAAGAG | 59.98 | 267 |
| 24 | TRINITY_DN115219_c0_g1_i1 | (AC)7 | ATTCACTTTCGCCAGTTGCT | 59.88 | ATTCTGAAATGGCCCTGTTG | 59.93 | 209 |
| 25 | TRINITY_DN118048_c0_g1_i1 | (CT)7 | CACAGCCCTAACCGTTGAAT | 59.99 | CTCCCAGAAAGCAGAAATGC | 59.96 | 197 |
| 26 | TRINITY_DN122314_c0_g1_i1 | (GA)6 | ACCACATGCAAGTCCATCAA | 59.97 | GCTTCTTGTTCTTCCTCCGA | 59.55 | 229 |
| 27 | TRINITY_DN123127_c0_g2_i1 | (CT)6 | ACAGAAACTGAGCCGGAGAA | 59.99 | TTCAATGCGGACATGTTTGT | 59.97 | 213 |
| 28 | TRINITY_DN12315_c1_g1_i2 | (AG)6 | GGGCATAATTGGCAGAACAT | 59.79 | GCCAAACAGTGGGTTTCCTA | 59.97 | 184 |
| 29 | TRINITY_DN12315_c2_g3_i1 | (CA)9 | AGCCTTTTGTCCCAGTGCTA | 59.88 | GCCCAAATGACCAAGGTTTA | 59.80 | 229 |
| 30 | TRINITY_DN12365_c0_g1_i6 | (AT)6 | AAAGAACCCCACTAGAACCCA | 59.85 | GGCAGGTTGTAATGCTGGAT | 59.96 | 257 |
| 31 | TRINITY_DN12371_c0_g1_i9 | (TC)6 | AGGACGGGACCAGTTACATC | 58.87 | GGTTTCGAACAGATCCCAAA | 59.91 | 265 |
| 32 | TRINITY_DN12375_c0_g1_i8 | (AG)12 | TAACGCAATCCCAACACAAA | 59.97 | GTATTCGTCTGCTCCGCTTC | 59.99 | 219 |
| 33 | TRINITY_DN128069_c0_g1_i5 | (TGG)6 | GGCTTCACTGTTGATTGGGT | 59.97 | CGTCTTCTCCAGAACTTGCC | 59.99 | 238 |
| 34 | TRINITY_DN131987_c3_g1_i1 | (CA)6 | AATCGACCGTACCAATCAGG | 59.81 | GTAGAGCCAAGCTTGATGGC | 59.99 | 181 |
| 35 | TRINITY_DN135432_c0_g1_i3 | (GAA)6 | CCACCGTTTCAGACAGCATA | 59.72 | AAAGGAGAAGGGGCAGAGAG | 59.95 | 252 |
| 36 | TRINITY_DN138617_c0_g3_i1 | (GA)8 | GCCTTTGAACATAGACCACCA | 59.99 | CTGGGTTGCTCATGCTTGTA | 59.86 | 235 |
| 37 | TRINITY_DN14371_c0_g1_i1 | (TC)9 | TAACATGTGCATTGGGAGGA | 59.92 | GGCAAAGAAGCGATTGTCTC | 59.96 | 205 |
| 38 | TRINITY_DN145189_c0_g1_i2 | (AAT)7 | GAAGAAGGCCTGCAAAAGAA | 59.57 | TTGTGGTGGTTGGTTCCTAA | 58.87 | 268 |
| 39 | TRINITY_DN148488_c0_g2_i1 | (GA)6 | GCAGGAGGTTAGGGATAGGG | 59.92 | TGACGGCATCTTCCTTCTCT | 59.95 | 246 |
| 40 | TRINITY_DN15022_c1_g1_i1 | (GTA)7 | CCTTCATGGTTTGGTCACCT | 59.82 | CCACATGTTAGATTCTTGAAGGAC | 59.07 | 274 |
| 41 | TRINITY_DN15052_c0_g1_i4 | (GCC)6 | GTCTGGTATTCGAGGCTTGG | 59.69 | AACAACAAGGCCCAATGAAC | 59.84 | 246 |
| 42 | TRINITY_DN15065_c0_g3_i4 | (TA)7 | TAGCCGCCTTTTCGTTCTTA | 59.98 | CTGATGCTGACGAAGATGGA | 59.94 | 194 |
| 43 | TRINITY_DN150955_c0_g1_i3 | (GA)7 | TAAACGCGGGATAGGAAGAG | 59.32 | CATCCACATTGGCTTCTTCA | 59.65 | 245 |
| 44 | TRINITY_DN15262_c1_g1_i4 | (GAG)7 | ACGGGATAGCGTCTCTTGAA | 59.84 | CATCACTCGTGTCGCATTCT | 59.86 | 188 |
| 45 | TRINITY_DN15284_c0_g1_i1 | (AGA)5 | TATTGCCACCACACATTGCT | 60.00 | ATTCCCCACGTACTTGGTTC | 58.77 | 229 |
| 46 | TRINITY_DN15284_c0_g2_i1 | (AAG)7 | CCCAGTACCTATTGCCTCCA | 59.95 | GCCAACGTTATTTGTTGTCG | 59.09 | 257 |
| 47 | TRINITY_DN154064_c0_g1_i1 | (AG)7 | GAGGCATTAGGGTTCCATCA | 59.89 | GGCTGGCAAACTTACCCATA | 59.96 | 211 |
| 48 | TRINITY_DN155879_c0_g1_i1 | (CTG)6 | TCAAAATCTAACCCGCCAAC | 59.94 | GGCGTGAGACTCGGTAACTC | 59.87 | 269 |
| 49 | TRINITY_DN159249_c0_g1_i1 | (AT)6 | TACAAAATGCAGGGAAAGGG | 59.93 | TCGTATGTGCTTGGGCTATG | 59.71 | 255 |
| 50 | TRINITY_DN16112_c0_g1_i4 | (CTT)7 | GAGTTTGTGCAGCAGATGGA | 59.99 | CGACAGCAACCATTCTTGAC | 59.29 | 268 |
| 51 | TRINITY_DN16127_c0_g1_i4 | (TA)6 | TCTTTTCCAGGAAGGTGCAT | 59.67 | TTCTGGGCAAATACCTCACC | 59.93 | 279 |
| 52 | TRINITY_DN162639_c0_g1_i1 | (TCT)5 | CTTCCTCTGGCTCCGTATTG | 59.83 | GAGAACGAGACCCCAGTGAG | 59.84 | 274 |
| 53 | TRINITY_DN163733_c0_g1_i1 | (AG)12 | ATGCGTTGTCATGAGCATTC | 59.69 | TCCAGCCTTACAGATGCAAA | 59.42 | 183 |
| 54 | TRINITY_DN166458_c0_g1_i1 | (CGC)5 | CGGAGTCCAGATTGAAGAGC | 59.95 | GAACAAGAACAAGGGCAAGC | 59.86 | 241 |
| 55 | TRINITY_DN167053_c0_g1_i1 | (GAC)5 | GGTTGAGTTCCCACCAAAGA | 59.94 | AAACTGGCCTTCCCAGAAAT | 59.94 | 229 |
| 56 | TRINITY_DN167146_c0_g1_i1 | (GA)6 | AGGAACGGAATGAGGGAGAT | 59.90 | GAGTGATTTGACGCCCTTTC | 59.68 | 275 |
| 57 | TRINITY_DN167264_c0_g1_i1 | (GA)7 | AGGATATGGAGTGTGACATAGCA | 58.62 | GGTCTGAAACTGCAGGAAGG | 59.84 | 247 |
| 58 | TRINITY_DN167351_c0_g1_i1 | (TGA)5 | GAGAGAAAGTTGGCGGTTTG | 59.85 | GCCAAGGCAACTTTCAAGAG | 59.99 | 275 |
| 59 | TRINITY_DN170911_c0_g1_i1 | (CTG)5 | TACCGCTCCGAGTCTCTTCT | 59.19 | AGCAGGAGGGACAGAGACAG | 59.58 | 218 |
| 60 | TRINITY_DN17241_c0_g1_i1 | (GA)7 | AGAGCTGCATGGGTTTCACT | 59.87 | TTTTGCTGGGTTTCAGCTTT | 59.86 | 244 |
| 61 | TRINITY_DN17322_c0_g1_i6 | (AG)6 | CTTTCTTCGTCCCCTTTCCT | 59.69 | AGAGCGTCGGATGCTAATGT | 59.87 | 200 |
| 62 | TRINITY_DN17361_c0_g1_i14 | (GGCTGC)6 | ACTGCGGGATGTAGGTCAAC | 60.00 | ATTTTCAGGGACAGGGCTTT | 59.94 | 244 |
| 63 | TRINITY_DN173732_c0_g1_i1 | (TC)7 | GAGCAAGGAGCTGGAATTTG | 59.96 | TGCTGTGCCACTAGGATACG | 59.89 | 244 |
| 64 | TRINITY_DN174841_c0_g1_i1 | (AC)7 | CTGAGCAAGTCAACATCCGA | 59.98 | CCCATTGGCGAAACTTGTAT | 59.82 | 239 |
| 65 | TRINITY_DN17497_c0_g1_i1 | (GAA)5 | GGTGAGGTGAAAAGAAACGC | 59.72 | GCAAAGCAAAGACTCGGAAC | 60.00 | 212 |
| 66 | TRINITY_DN176078_c0_g1_i1 | (CT)6 | GAGAGAACCGTCCTGCACTC | 59.99 | AGAAACTCAGGCCGTTGAGA | 59.99 | 240 |
| 67 | TRINITY_DN177283_c0_g1_i1 | (CTT)5 | AAGGGTGTGTGGCAATAAGC | 60.00 | AGAATTTCCGAGACGCAAAA | 59.82 | 251 |
| 68 | TRINITY_DN177708_c0_g1_i1 | (GTT)6 | CTTTCTCCATCTGGGCTGAG | 59.94 | TGCGGACAAGCTAGAGGATT | 59.98 | 207 |
| 69 | TRINITY_DN17797_c1_g1_i11 | (AT)10 | TGAGTTCACCTGCAATCGAG | 59.98 | CATTCTGCGAACCTGTTTCA | 59.84 | 280 |
| 70 | TRINITY_DN18137_c0_g2_i1 | (CCT)8 | GCAGGACAGAGCGATTCTTT | 59.58 | AGCAAAGGTGGCTGTGAAGT | 59.91 | 267 |
| 71 | TRINITY_DN18196_c1_g1_i1 | (TA)9 | TCCTTTCTCCCCAAACACAC | 59.94 | CACTAATGTGGCCTGGTGAA | 59.57 | 236 |
| 72 | TRINITY_DN18215_c0_g1_i2 | (AG)6 | GCCACCACAAACCTTCATCT | 59.97 | GCCCAGAGACTTCTCCCTCT | 59.95 | 232 |
| 73 | TRINITY_DN185046_c0_g1_i1 | (GA)11 | AGGGCCGACCAAAAGAGTAT | 59.96 | TTCTCTTTCGGCAACTTGGT | 59.85 | 279 |
| 74 | TRINITY_DN186588_c0_g1_i1 | (TC)7 | CGGTTTGGGTGCTCTCTATC | 59.69 | GGTGGTCAGTGACGGAGATT | 59.97 | 234 |
| 75 | TRINITY_DN189572_c0_g1_i1 | (TA)7 | ATTGATGAGGCTCCGAGAGA | 59.91 | AGGAGGCAAGGATAACAGCA | 59.84 | 234 |
| 76 | TRINITY_DN189720_c0_g1_i1 | (CT)11 | AGAAGTGATGAACCGCTCGT | 59.87 | GGTGCGTGACAGTATTCTCG | 59.32 | 185 |
| 77 | TRINITY_DN189766_c0_g1_i1 | (GT)7 | AGGATCGACCAGTCAACAAAG | 59.20 | CACACCAAGACATGGACAGG | 60.00 | 279 |
| 78 | TRINITY_DN2010_c0_g2_i16 | (GGCACA)6 | GAGACCATCGGCTCAGTTTC | 59.81 | GCAGTCAAACTCATCCAGCA | 59.99 | 220 |
| 79 | TRINITY_DN2012_c0_g1_i1 | (AT)12 | ATCAATAGTTGCCCCAGCAC | 59.96 | ATCAATAGTTGCCCCAGCAC | 59.96 | 246 |
| 80 | TRINITY_DN201747_c0_g1_i1 | (TC)10 | CTCTGTCCTTCTCCCTCCCT | 59.80 | GAGGGAACAAGGAAAATCCC | 59.74 | 203 |
| 81 | TRINITY_DN2018_c1_g2_i3 | (AAG)6 | ATGAGGCCAGGATACACAGG | 59.95 | CCAAGGTTCCACGTCATCTT | 59.97 | 260 |
| 82 | TRINITY_DN203_c0_g1_i1 | (TC)8 | GAGATGAGTGGGAGCTTTGC | 59.96 | GGCTTGTGATCCGAAATCAT | 59.90 | 273 |
| 83 | TRINITY_DN203_c0_g1_i1 | (GA)8 | GATGCGTCTCACTGCATCAT | 59.83 | CTTAACAACGTCGTCTGCCA | 59.90 | 226 |
| 84 | TRINITY_DN2039_c1_g1_i1 | (AGT)5 | TGGCGAATTTATTTGGAAGG | 59.90 | CCTCGTCCACTTCCATTGAT | 59.93 | 249 |
| 85 | TRINITY_DN2039_c1_g1_i25 | (ACT)5 | CCTCGTCCACTTCCATTGAT | 59.93 | TGGCGAATTTATTTGGAAGG | 59.90 | 249 |
| 86 | TRINITY_DN2041_c0_g1_i11 | (TC)15 | ATTCGCTCTGCACATGTTTG | 59.87 | CAGGGGCTTTTACTTGACTGA | 59.36 | 203 |
| 87 | TRINITY_DN2045_c0_g1_i2 | (AT)12 | GTCCAAACGCAATGCCTAAT | 59.97 | ACTTTCGCAGCACAACCTTT | 59.92 | 185 |
| 88 | TRINITY_DN205740_c0_g1_i1 | (CTC)5 | CGCATACCTTGCAGACAAAA | 59.87 | AAGGTGGGGTCAGTGATGAG | 59.96 | 254 |
| 89 | TRINITY_DN207_c0_g1_i9 | (GA)19 | TTGAAGGTCTGCTGTTCTGG | 59.01 | GGCATGTTCAAGCCACCTAT | 59.96 | 263 |
| 90 | TRINITY_DN207_c2_g1_i1 | (ACC)5 | CCGCTCCTAGATTGCGTAAG | 60.00 | GGCTTGTGCATTCCTAGCTC | 59.99 | 259 |
| 91 | TRINITY_DN208509_c0_g1_i1 | (GA)10 | TGAAGGCAAGCAAGATTTGA | 59.54 | TGGTTTGTGTATGCCCTCAC | 59.42 | 246 |
| 92 | TRINITY_DN2090_c0_g1_i24 | (CT)8 | AAATGCGACAGGCAAGAAGT | 59.88 | CGTTCTTGAGAGGGCTTGAC | 59.99 | 248 |
| 93 | TRINITY_DN209164_c0_g1_i1 | (CT)6 | TGTTGGGACTTTTCCTGGAT | 59.38 | GAGGTAGCTAGAGGGGCGTT | 59.87 | 193 |
| 94 | TRINITY_DN209630_c0_g1_i1 | (TC)7 | GTGGGAGAGTTGGAGCTCAG | 59.99 | CACTGGGATCATCATTGTCG | 59.92 | 248 |
| 95 | TRINITY_DN212279_c0_g1_i1 | (TA)6 | TAGCCACCAATCCCACTTTC | 59.93 | AAGGCGGTGTCTTTCTCTGA | 59.99 | 265 |
| 96 | TRINITY_DN21414_c0_g1_i1 | (AC)9 | TGATGTTTCCCACAGATCCA | 59.89 | CTCTGCTTTCGGGTCAGTTC | 59.99 | 262 |
| 97 | TRINITY_DN216519_c0_g1_i1 | (AG)6 | CAGGTGAAACGGAAGTCAGA | 58.85 | ATACGGAGGTGGTTCACGAG | 59.99 | 182 |
| 98 | TRINITY_DN21713_c2_g2_i1 | (AT)6 | CTTGTGGTAAACCCTGGGAA | 59.82 | GCAATGCCAAGTGCAACTAA | 59.88 | 279 |
| 99 | TRINITY_DN219_c0_g1_i2 | (TCC)7 | ACCTCTCTTGCCCACTTTCA | 59.84 | GGGGTGAAAACATCGAGAAA | 59.91 | 195 |
| 100 | TRINITY_DN2215_c0_g1_i38 | (CT)9 | GCCAGTGCATCTTGTTGGTA | 59.72 | TGCCAGCACTGTTAATGAGG | 59.86 | 268 |
| 101 | TRINITY_DN2241_c2_g1_i12 | (GA)6 | TGAGAGGGAGCATGCATAAA | 59.38 | GGGGCTAGGAGCTTAAGGAA | 59.81 | 260 |
| 102 | TRINITY_DN225_c0_g1_i21 | (CGA)8 | CGGCTTTATTGACGAGAAGC | 59.98 | TATCCGTACCACCATGCAGA | 59.95 | 278 |
| 103 | TRINITY_DN2252_c0_g1_i4 | (CA)9 | GCCAGTGAAAGAACGCTTATG | 59.90 | GCTTTCCTCGCGTATGTTTT | 59.37 | 210 |
| 104 | TRINITY_DN2253_c0_g1_i1 | (TGC)8 | TCTTTAGCCTTGCCTTCCAA | 59.95 | TATGCAGCAGCAGAACCATC | 59.98 | 209 |
| 105 | TRINITY_DN2253_c0_g1_i22 | (CTG)5 | CGTGTATCAGGCGGAGGTAT | 59.98 | GCAACATTACGTGCAGAGGA | 59.87 | 267 |
| 106 | TRINITY_DN2281_c0_g1_i35 | (AG)6 | TCGCAACGCATTAAGAGAGA | 59.71 | CAGCACTCGTTTCCTTCCTC | 59.99 | 218 |
| 107 | TRINITY_DN2293_c0_g1_i13 | (GT)7 | ATTTGAGGCGTTTTGGTTTG | 59.98 | TTCTGCGACATCATGAAAGC | 59.96 | 274 |
| 108 | TRINITY_DN2295_c0_g1_i1 | (GA)7 | CGCCACCAAAGATTTTCAAT | 59.94 | TCTCCAGAAGCTCCACTGGT | 59.99 | 277 |
| 109 | TRINITY_DN240_c0_g1_i2 | (GA)8 | TGGCTCCTTGAGAGACCTGT | 59.99 | CAGGCTTCCTCTCTGTGACC | 59.99 | 184 |
| 110 | TRINITY_DN256_c0_g1_i4 | (GA)11 | GTTTCGACTTGAGGGGAGAG | 58.87 | GAGAAAGCCTGCCAGAGAAA | 59.69 | 268 |
| 111 | TRINITY_DN264_c5_g1_i1 | (AAG)7 | AAATCCACATCCGCAAGTTC | 59.94 | AGACAACGCAAATCAATCCC | 59.94 | 222 |
| 112 | TRINITY_DN267_c5_g1_i1 | (AT)6 | GGTCGGGTCTTAATGAAGGAT | 59.30 | GGGTGTTAAAGCAGCCCATA | 59.96 | 271 |
| 113 | TRINITY_DN26716_c1_g2_i1 | (TC)7 | TCTCAAGCACCAGCTTTTCA | 59.72 | CATCGCAAGTGGGAACAGTA | 59.72 | 188 |
| 114 | TRINITY_DN2691_c2_g4_i1 | (GA)9 | CCAACAACTCCACAAACCCT | 59.86 | TTGTAAACCATCAACCCTACCC | 59.98 | 256 |
| 115 | TRINITY_DN2691_c3_g1_i2 | (TC)6 | TATTTCGCCCCAATTGTCTC | 59.90 | TACGGGTATGACATTCGCAA | 59.95 | 240 |
| 116 | TRINITY_DN28142_c0_g1_i1 | (A)10 | GAAGTTATCCCGATGCCAAA | 59.90 | TTTACCACTTGAGCCAACCC | 59.97 | 212 |
| 117 | TRINITY_DN2816_c4_g1_i5 | (GCA)6 | TTTCGGAGCGGTTCTACAAT | 59.71 | GACAACGGGAGTGAACGATT | 59.97 | 248 |
| 118 | TRINITY_DN2826_c0_g1_i2 | (TA)7 | ATGCAAACTGGAAAGGAAGC | 59.32 | ATCGTCGAGGAGGATTCAGA | 59.76 | 276 |
| 119 | TRINITY_DN2844_c0_g1_i2 | (AG)6 | TTGAAAGCAATTCACGGATG | 59.66 | GCAAACGACAAGCAGATCAA | 60.00 | 247 |
| 120 | TRINITY_DN2848_c0_g1_i8 | (CT)15 | CAACTCGTACGTGCGATCAT | 59.75 | AGTGAGAGCTTGGAGCTTGG | 59.75 | 228 |
| 121 | TRINITY_DN2856_c0_g1_i11 | (GA)9 | GACGCAGCAATGAAGCATAA | 59.98 | TCTCAGCCTCACCACCTTCT | 59.99 | 229 |
| 122 | TRINITY_DN2857_c0_g1_i5 | (CTA)5 | GGGTTTTCAGAAACCGTTGA | 59.95 | CCACGATGTTCTTTCGGAAT | 59.93 | 208 |
| 123 | TRINITY_DN2885_c1_g1_i1 | (TCT)5 | AGCCAATGTCGGAGAGAAGA | 59.95 | TCAGGGAGAAAGAGAACCGA | 59.92 | 233 |
| 124 | TRINITY_DN28989_c0_g1_i1 | (AG)18 | TGCTCGTTTGCTGTAACAATG | 59.93 | ATCTACGAACCGGTGCAAAC | 60.00 | 187 |
| 125 | TRINITY_DN296_c0_g1_i8 | (TC)8 | TCCCTGGTGGAGATGAAAAC | 59.90 | AGAACATCAAAGGGCCAGAA | 59.67 | 241 |
| 126 | TRINITY_DN297_c0_g2_i1 | (GA)7 | GCAGACACTGATGGAAGCAA | 59.99 | AATCCAGAACGGTAGCCAAT | 58.53 | 239 |
| 127 | TRINITY_DN29711_c0_g1_i1 | (TC)11 | CTTGGTCCTTTTTGCTTTGG | 59.72 | CGGAAAAGAAAGCTCGAGAA | 59.70 | 243 |
| 128 | TRINITY_DN29746_c0_g1_i9 | (AC)13 | AGGAGATGCTCTTGATGCGT | 59.98 | CTGCGACAAAGTGCAAATGT | 59.91 | 207 |
| 129 | TRINITY_DN3001_c0_g1_i4 | (AG)7 | GCGTTCGACTTCCCTTACAG | 59.88 | ACAACAGGTCAATGGCTGTG | 59.60 | 196 |
| 130 | TRINITY_DN3027_c0_g1_i6 | (CT)6 | CAGGTGTGGCATGGATGTAG | 59.98 | CTGTTTTCCATTGCTTGGGT | 59.97 | 250 |
| 131 | TRINITY_DN3037_c0_g1_i1 | (GA)7 | TCAGACCCAAGCAGAGGAGT | 59.99 | AGAAGATGTGCGAGGAGACC | 59.41 | 242 |
| 132 | TRINITY_DN3040_c0_g1_i1 | (GCA)5 | AGGCAGTTAAGCAGAATGGG | 59.34 | AACCAACCAACCAACCACAT | 59.99 | 264 |
| 133 | TRINITY_DN3057_c0_g1_i5 | (CT)15 | ATGGGGAAGCTCAAAAGGAT | 59.90 | CCTAACTCACAGGTGCACACA | 59.80 | 270 |
| 134 | TRINITY_DN3062_c0_g1_i2 | (AT)6 | GACAGCTCCTGCAGTCAATG | 59.58 | GAAAATCCACGGCTTGTAGC | 59.71 | 260 |
| 135 | TRINITY_DN3063_c0_g1_i3 | (TC)8 | GGTTGGTACATCGGCATTCT | 59.82 | AACAGATGTCCCCTTTGACG | 59.97 | 276 |
| 136 | TRINITY_DN3064_c0_g2_i2 | (TC)9 | TTGTTGCGAAAATTGTGCTC | 59.86 | CCACCCTACAACAACGACAA | 59.46 | 275 |
| 137 | TRINITY_DN3084_c0_g1_i6 | (TC)8 | TCAAACACTCTCTTCAAACCCA | 59.75 | CTCCTCCAGCTGGTTAAACG | 59.87 | 198 |
| 138 | TRINITY_DN3096_c1_g3_i1 | (GA)8 | AACGATTACGCGGCATTTAC | 59.99 | TCAATTCTGGCTTCAACTTCTG | 59.49 | 274 |
| 139 | TRINITY_DN32163_c0_g1_i1 | (GA)11 | TACGCTCGCTGTCAAGCTAA | 59.92 | TGCCGAGTCCAGAATCCTAT | 59.65 | 186 |
| 140 | TRINITY_DN33010_c0_g1_i1 | (GAA)5 | TCCTCTTCTGTTTCCGCATT | 59.81 | GTGCTTCATCAACTTTGCCA | 59.85 | 265 |
| 141 | TRINITY_DN3323_c1_g1_i18 | (GA)10 | CGTTGGATAAGCAGGTCCAT | 59.96 | CCTCAAGTTTCTGCCTGGAG | 59.98 | 258 |
| 142 | TRINITY_DN3323_c1_g1_i18 | (AG)12 | TGTGTATGTGTACGGGAGTGG | 59.35 | ATGGACCTGCTTATCCAACG | 59.96 | 225 |
| 143 | TRINITY_DN3334_c0_g1_i13 | (TC)9 | CTTGGCTGCATGTGTGAAGT | 59.91 | GCCGAATCGTTCTGAGAGTC | 59.96 | 181 |
| 144 | TRINITY_DN3334_c0_g1_i7 | (CT)15 | GGGTCTTCGCTTCTATGTGC | 59.84 | GTCGTTCTGGGAGTCGAGAG | 59.99 | 222 |
| 145 | TRINITY_DN36043_c0_g1_i2 | (AC)7 | ATGTCCCCATTGTTCCCATA | 59.87 | GTTTTCGTTGCTGGTTGGAT | 59.98 | 256 |
| 146 | TRINITY_DN3615_c1_g1_i52 | (AT)12 | TCACGCCATAACGTTAACCA | 59.99 | TCACGCCATAACGTTAACCA | 59.99 | 206 |
| 147 | TRINITY_DN3620_c0_g1_i10 | (GA)15 | TCTCTTGCGCTTTGTTTTCA | 59.73 | ACTGATTCGTTTCACTCGCA | 59.45 | 221 |
| 148 | TRINITY_DN3620_c0_g1_i4 | (AG)16 | CAGCGTCTCCACTCACAGAG | 59.76 | TTCTTGATCTGGCTGAGGGT | 59.80 | 246 |
| 149 | TRINITY_DN3620_c2_g1_i5 | (CT)7 | CCTTTCTCTTGGGCACTCAG | 59.98 | CACACACAAACGCACAGAGA | 59.48 | 214 |
| 150 | TRINITY_DN3633_c3_g1_i4 | (AG)15 | GGTTTTCTTGCATTTTGCTG | 58.41 | GCTTTGGTTTCAATTTGGCT | 59.21 | 277 |
| 151 | TRINITY_DN3637_c0_g1_i1 | (AT)12 | TAAGGGAAAGCAGGGGAAAT | 59.90 | CCTACCTGTGGGAAATCGAG | 59.55 | 236 |
| 152 | TRINITY_DN3637_c0_g1_i5 | (AT)12 | CCAAAACCCTTCTTGTGCAT | 59.97 | GATGGTTAGCAAGCCTCTGG | 59.84 | 268 |
| 153 | TRINITY_DN3640_c0_g1_i44 | (CT)18 | CTCCGACTTCTCTGGTTTCG | 59.98 | GACTCTGGGAAGGGGATTTC | 59.87 | 259 |
| 154 | TRINITY_DN3649_c0_g2_i3 | (CTT)5 | ACCATAGAGTCCGCCTTTCA | 59.69 | CCTCCACTACCACCACCACT | 59.88 | 230 |
| 155 | TRINITY_DN3656_c1_g1_i17 | (GA)15 | TTCTCCGTGTACTTCCACTGTC | 59.26 | GCCCACCATGTTCTAATCGT | 59.82 | 200 |
| 156 | TRINITY_DN3670_c1_g2_i2 | (GA)7 | GCCGTTGGATTGCATCTTAT | 59.93 | TCCTCTTGCCTTTGGAGTGT | 59.84 | 193 |
| 157 | TRINITY_DN3686_c0_g2_i1 | (TC)15 | GCCGTTTAAGCAGAATGAGC | 59.99 | AGAGAACCCTGGACGAGACA | 59.84 | 211 |
| 158 | TRINITY_DN3691_c0_g1_i13 | (AT)9 | GGATCAGATGAAGACACGTCAG | 59.73 | AAAAGGACACCCAGATTTGGTA | 59.74 | 185 |
| 159 | TRINITY_DN3694_c0_g1_i1 | (TC)6 | CGCTCACGACTGCAAATATC | 59.45 | CACGAGTGTGTGTCAGCGTA | 59.51 | 198 |
| 160 | TRINITY_DN3695_c0_g2_i20 | (AG)12 | CAAAATCCACGTCTCCACAA | 59.54 | CTCAGCAGCAATGACGAGAG | 59.88 | 239 |
| 161 | TRINITY_DN3908_c1_g1_i2 | (AG)10 | AGCAACCCATATGTGAAGGC | 59.96 | TAGCCCGGAACGTAGAGAGA | 59.97 | 265 |
| 162 | TRINITY_DN3924_c1_g1_i2 | (AG)6 | GGGAGTGTGCGTGGATCTAT | 59.96 | TAGCCACCCATGGGAGTAAG | 59.95 | 249 |
| 163 | TRINITY_DN3925_c0_g1_i23 | (CAG)7 | GGAGAAAGGCCACCAACATA | 59.93 | ACCTCCTGCTTCTGCATTGT | 59.87 | 225 |
| 164 | TRINITY_DN3933_c0_g1_i4 | (ATC)5 | ATCGAAACAAATCCATTCGC | 59.91 | TTTAACCACACGCAGTCTCG | 59.90 | 199 |
| 165 | TRINITY_DN3935_c0_g1_i4 | (CT)9 | ATTTTGCAGCACATTCGTTG | 59.74 | ATTGACGTACCGTGCATTGA | 60.00 | 238 |
| 166 | TRINITY_DN3941_c0_g2_i4 | (AG)18 | GTGCGTGAACGTGATGAACT | 59.76 | ATCACGCAGAAAAGCCACTT | 59.88 | 238 |
| 167 | TRINITY_DN3954_c0_g1_i1 | (GT)10 | TTGGCTGTCTGCGATTTTAG | 59.06 | ATCCTTCTTCCTGGCTGTCA | 59.80 | 270 |
| 168 | TRINITY_DN3972_c2_g1_i2 | (CT)10 | CCAGTTCCAGCTATTTTCGC | 59.85 | ATGACCAGGACCAGAAGTGG | 59.96 | 273 |
| 169 | TRINITY_DN3983_c0_g2_i1 | (GAG)7 | CTTCAGGCAGAGGAAGATGG | 59.94 | GTGTTCTCGCTCTGTTGCTG | 59.78 | 225 |
| 170 | TRINITY_DN3984_c0_g1_i9 | (TC)25 | ATTGCCAAGGAGGTGATGTA | 58.02 | GCATGTTCGACTTGACTGGA | 59.84 | 264 |
| 171 | TRINITY_DN4210_c1_g1_i17 | (CT)6 | CCCAACCGCTGTATTTCTGT | 59.99 | CAGGGCATCATTCCAAAACT | 59.93 | 240 |
| 172 | TRINITY_DN4256_c0_g1_i37 | (TC)6 | GTCTCACTCCACGCTTCTCC | 59.99 | TCTCGCTTTCCAGTCAGGTT | 59.99 | 202 |
| 173 | TRINITY_DN4273_c0_g1_i6 | (TA)6 | AGTTTGTGCGGATTTGGTTC | 59.98 | GAGCATCAACCTCCCAACAT | 59.93 | 271 |
| 174 | TRINITY_DN4280_c0_g1_i2 | (CT)7 | TTTTCCCTCACTTTCTCGGA | 59.78 | GACGACGGAGCAGAGAAAAC | 60.00 | 253 |
| 175 | TRINITY_DN44949_c0_g1_i1 | (TA)11 | CTGACCCGACCCACTAATGT | 59.84 | TCCAAATCGGACTCTGATACC | 59.00 | 220 |
| 176 | TRINITY_DN4616_c1_g1_i2 | (CT)7 | GGACGATTGCAAAAGAGAGG | 59.81 | GGAAGCCATGAAAGGAGCTA | 59.41 | 260 |
| 177 | TRINITY_DN4621_c0_g1_i1 | (TAA)5 | TTGCCGACAATTCCTTCTCT | 59.81 | GCTGTCCAAGACAAGCATGA | 59.99 | 233 |
| 178 | TRINITY_DN4682_c0_g2_i1 | (AAG)6 | GGTCGCAATCATAGTCCGAT | 59.92 | TGGTCATTGCGTGATGTTTT | 59.97 | 205 |
| 179 | TRINITY_DN5101_c0_g1_i7 | (AG)6 | AACCAAACAAGCCATGAAGG | 59.97 | AGCCCTCAAAGATTGCAAAA | 59.82 | 202 |
| 180 | TRINITY_DN5108_c0_g2_i1 | (GGT)6 | GACCCCAAGACAAGTCGAAG | 59.70 | ATCATCATCCGACGACAACA | 59.93 | 268 |
| 181 | TRINITY_DN5134_c0_g1_i2 | (TC)7 | GTTTTCACCGGACGAGTCAT | 59.97 | CAAACCCCTTCAGAAACCAA | 59.94 | 238 |
| 182 | TRINITY_DN5137_c0_g1_i17 | (CT)12 | CCTCTCCTTCATCCTGTTGC | 59.80 | GGACCTTCCATTGGGGTATT | 59.88 | 279 |
| 183 | TRINITY_DN5137_c0_g1_i9 | (CT)12 | AAAATCACCTTGGATTTGCG | 59.94 | TCCTCTTTAGCTGGTTCCCA | 59.81 | 275 |
| 184 | TRINITY_DN5138_c0_g1_i2 | (TC)11 | TGCTTTGCTTTTCTCCCTGT | 59.99 | ATGGAGCTTTCACTGCGTTT | 59.88 | 243 |
| 185 | TRINITY_DN5177_c0_g2_i2 | (AT)8 | CAGGAATGAACACGGCAGTA | 59.72 | CCTGGATTTTGTTCCTCTGC | 59.67 | 236 |
| 186 | TRINITY_DN5182_c0_g1_i7 | (GAG)6 | CCTTGCATTTTACACCAGCA | 59.73 | GCTGATTTCCACGTTGGTTT | 59.98 | 249 |
| 187 | TRINITY_DN5193_c1_g1_i1 | (TC)15 | GACCTCAAACCAGATCTTCCA | 59.13 | CCAGTTCCCAGGTGATGTCT | 59.96 | 264 |
| 188 | TRINITY_DN5312_c2_g1_i12 | (CTG)5 | AGAGGAGGGAAAGGCTTCAG | 59.95 | CCCTGAACACCAAAGCAAAT | 59.97 | 221 |
| 189 | TRINITY_DN5316_c0_g1_i2 | (CTC)7 | TCCATTCTGTTCCCTTCCAC | 59.90 | GAAGAGGCAAGTGGAAATCG | 59.81 | 186 |
| 190 | TRINITY_DN5321_c0_g1_i18 | (GCT)6 | GGGCTTCTGTTATGGTGCTC | 59.70 | TCCTCCTCATGTGCTCTGTG | 59.98 | 253 |
| 191 | TRINITY_DN5382_c1_g1_i3 | (TC)7 | CGGAAAAGAGACCTGAATGC | 59.81 | TTCGTTTCTGTTCCACGAGA | 59.42 | 211 |
| 192 | TRINITY_DN5517_c0_g1_i2 | (TA)6 | TAAGAGCGAAGGTCGTGGTT | 59.88 | GACTTGTGTCCTGAATGCGA | 59.84 | 222 |
| 193 | TRINITY_DN5534_c0_g3_i2 | (GA)9 | AAGGCTTGCACCTCCTAACA | 59.88 | GTTCCACATGATCCATTCCC | 60.00 | 240 |
| 194 | TRINITY_DN5563_c1_g2_i3 | (AT)8 | CAAATACTCCCGTTCCTCCA | 59.93 | AAAAGAGTTGAGGCGGCATA | 59.85 | 261 |
| 195 | TRINITY_DN5577_c0_g1_i1 | (TC)12 | GACCAGGGTACAGGGTCTCA | 59.96 | CCCACATCGGTAGCAAAAGT | 59.99 | 209 |
| 196 | TRINITY_DN5577_c0_g1_i5 | (CT)10 | ATGGTGGTGAGGAGGAACTG | 59.96 | TACCTTTATCCGCCCATCTC | 59.00 | 220 |
| 197 | TRINITY_DN5577_c0_g2_i19 | (CT)8 | TTTGGCACTTGTGTTTCTGC | 59.89 | CTTGCCGATACAAACCGAAT | 59.96 | 268 |
| 198 | TRINITY_DN56766_c0_g1_i4 | (GA)8 | GCGATGGACCTTGTGTATGA | 59.53 | GCGTGGTCCAGTCCTTGTAT | 60.00 | 202 |
| 199 | TRINITY_DN56782_c0_g1_i1 | (CT)9 | GCCTTGTACACCTTCCCGTA | 59.99 | AAACCTCCTCCCCTCAAAAC | 59.42 | 215 |
| 200 | TRINITY_DN5701_c2_g2_i1 | (TA)6 | TTGAATGTTGCAACCGATGT | 59.97 | TGGATGAACAGTGTTGGCAT | 59.97 | 220 |
| 201 | TRINITY_DN5716_c0_g1_i5 | (TC)14 | ATGAGATGGTTGCCCAAAAC | 59.80 | GGACTTCATTTGGCCCTGTA | 59.93 | 193 |
| 202 | TRINITY_DN5739_c0_g3_i1 | (CTT)5 | TCAATCTCTGTGCGGCTATG | 59.97 | CCAACGGAGAAGAAGAATCG | 59.81 | 276 |
| 203 | TRINITY_DN5764_c2_g1_i2 | (AAAAT)5 | AGGGACCATCCTGTTGTTTG | 59.82 | TGGAATCAGCCGTATGTTTG | 59.54 | 181 |
| 204 | TRINITY_DN5765_c0_g1_i14 | (AT)8 | GCCATGAGAAGTGTCAAGCA | 59.99 | TCAACGGAAGTGTGAGTTCG | 59.87 | 270 |
| 205 | TRINITY_DN5793_c8_g1_i1 | (AG)9 | TCACATATGGTCCTCTTCTCCA | 59.55 | AAGAGCCCTTGTAGAACTCTCG | 59.19 | 181 |
| 206 | TRINITY_DN59271_c0_g1_i1 | (GA)6 | CTCCCGGAACACAATCAAAT | 59.79 | TGAACCCAAAGGTCCAGAAC | 59.94 | 226 |
| 207 | TRINITY_DN59996_c0_g1_i1 | (AG)7 | TCCCAAATCCAAAACTAGCA | 58.21 | CACTCTCATCGCAAGCACTC | 59.73 | 228 |
| 208 | TRINITY_DN6233_c0_g1_i6 | (TCA)5 | ATAAACGACACGCGACACAC | 59.65 | GAACTTGTGCTGCCTGATGA | 59.99 | 274 |
| 209 | TRINITY_DN6239_c0_g1_i1 | (GA)11 | GAGCGAAAACTGTGCCTTTC | 60.00 | TTGGCTAGAGGTTGAAGGGA | 59.81 | 248 |
| 210 | TRINITY_DN6260_c0_g1_i1 | (CT)8 | TTCGCTGCTTCCTCTCTCTC | 59.97 | TCGTGTTGTCCGTTCTCATC | 59.68 | 250 |
| 211 | TRINITY_DN6286_c0_g1_i16 | (TA)12 | TGGTTGGATGTTTTGTTGGA | 59.79 | TGGCTCAAGGGAGGAAACTA | 59.81 | 210 |
| 212 | TRINITY_DN6308_c0_g3_i10 | (GGA)5 | CAGTGGGGAAGTCCCATAGA | 59.92 | GGTGCCTTTCGATTGGTAAA | 59.94 | 254 |
| 213 | TRINITY_DN6332_c0_g1_i19 | (AT)12 | CAGCATGGATTTTGACGAGA | 59.80 | TTCAACATCATGCACCCACT | 59.97 | 216 |
| 214 | TRINITY_DN6385_c1_g2_i19 | (TC)9 | ATTCGGTGCTACGATTTTGG | 59.96 | TCCTGGTCCTCAGCTGAACT | 59.99 | 238 |
| 215 | TRINITY_DN6602_c0_g1_i17 | (TG)8 | TGGGGTGTTGATACACAAAGA | 58.89 | CCTCAGCAACAGTCCCTTTC | 59.84 | 272 |
| 216 | TRINITY_DN6613_c2_g1_i1 | (AT)8 | AGCATTAGAAGGCAAGGCAA | 59.98 | CGTGTGGTAGCAGCAGAGAG | 59.79 | 269 |
| 217 | TRINITY_DN6674_c0_g1_i1 | (GGA)5 | AGTGATCGTGGGAAAGTTGG | 59.97 | ACATCTTCCCGTCACCAAAG | 59.97 | 181 |
| 218 | TRINITY_DN72062_c0_g1_i1 | (AT)6 | AGCATGGGTCCAAAATAAGC | 59.05 | GGGCTTCCATTGTTTGTCTT | 59.03 | 261 |
| 219 | TRINITY_DN7778_c0_g1_i5 | (GA)12 | ATGTTTCCCCGACTTTGTTG | 59.83 | CTGATCTTCAAGGCTTTGCC | 59.96 | 266 |
| 220 | TRINITY_DN7780_c0_g1_i1 | (TA)8 | TTGGATGAGGAAATCGGAAC | 59.87 | CGTTTCCGAGTCCTGGTAAT | 59.05 | 190 |
| 221 | TRINITY_DN806_c0_g1_i5 | (AAG)12 | ATTTCATGTGCTCCCCAAAG | 59.93 | CTTCTCTTGAGGCAGTCGCT | 59.89 | 213 |
| 222 | TRINITY_DN814_c2_g1_i1 | (CT)12 | CTAATGGTTGGTGTGCCAGA | 59.57 | GAACCACAAGGATTCCCAGA | 59.90 | 182 |
| 223 | TRINITY_DN816_c2_g1_i2 | (TA)6 | TCCCACAATTAACAAGTGCG | 59.59 | TCTTTCAGCTCGGTCATGTG | 59.98 | 240 |
| 224 | TRINITY_DN8300_c0_g1_i12 | (GT)12 | GATGACTGGCGCTAGAAAGG | 59.98 | TATGCCCCACAATCAGATCA | 59.88 | 249 |
| 225 | TRINITY_DN831_c2_g2_i1 | (TC)8 | CGGGATCTGAATGTGGTTTT | 59.79 | ACTGAGCTTGGGAAGGTGAA | 59.84 | 240 |
| 226 | TRINITY_DN8331_c1_g1_i4 | (TGC)5 | GGCAGGCTAAAATGCTGAGT | 59.48 | TCCATCTGGTTCACACTTGC | 59.68 | 279 |
| 227 | TRINITY_DN8335_c0_g1_i55 | (AT)8 | AATGACCTTCGCAATTCCAC | 59.94 | ACTTATTTGCCAACCGTCGT | 59.50 | 190 |
| 228 | TRINITY_DN836_c1_g1_i37 | (TG)6 | TGAAAGCGTGAAATGCAGAC | 60.00 | ATAATTTGCTGCCGTTTTGG | 59.96 | 237 |
| 229 | TRINITY_DN8391_c0_g1_i5 | (TA)12 | GGTATGCGGTGTAAAAGCCT | 59.11 | TCAATGTCGTGTCTCCAAAA | 57.64 | 259 |
| 230 | TRINITY_DN849_c2_g1_i3 | (CT)11 | GGTGTGCATCCAGGAGAAAT | 59.93 | CAGTGTGCGATCGTCTTTGT | 59.91 | 190 |
| 231 | TRINITY_DN849_c3_g1_i6 | (CT)11 | TCTTTGGTTTCCTTCCTCCA | 59.64 | AGCAAAGACGTTCCGAGAAA | 59.99 | 258 |
| 232 | TRINITY_DN856_c0_g1_i1 | (TGC)5 | ATGCGGAGTGGACCAGTTAC | 60.00 | AGAACAAAGCCCTGAAGCAA | 59.99 | 262 |
| 233 | TRINITY_DN861_c0_g1_i4 | (TC)15 | AGACTCGAAGGCTCTGCTTG | 59.89 | TATGGCACCTTTGTGGTCAA | 59.96 | 206 |
| 234 | TRINITY_DN8862_c1_g1_i2 | (TTC)6 | TTGACCTGGTAACCCGAATC | 59.79 | CTGCTTTTGGAGATCTTGCC | 59.96 | 248 |
| 235 | TRINITY_DN8862_c1_g2_i1 | (TCT)6 | ATCGTACCGTTGCTTGATCC | 59.96 | GATTCAGACGGGTCGTGTTT | 59.97 | 249 |
| 236 | TRINITY_DN8870_c0_g1_i9 | (AAG)6 | TGAAACTCATCCTTTCTTCCG | 59.30 | TCAAAGGCAAGTGCTGATTG | 59.99 | 245 |
| 237 | TRINITY_DN888_c2_g1_i2 | (GA)16 | CTTCGCCAGTCTACCTTGCT | 59.64 | CAGTCATGCTTCTCCAACGA | 59.98 | 221 |
| 238 | TRINITY_DN888_c5_g1_i1 | (GGT)6 | CGTTGGTATAACCTGTTGCG | 59.11 | TCCTTCAAGTCCTTCTGCGT | 59.99 | 215 |
| 239 | TRINITY_DN889_c0_g1_i25 | (TC)9 | CCAGCCACTCTCGTCTCTCT | 59.73 | TACTGGGTCAGGGTGAGGTC | 59.96 | 245 |
| 240 | TRINITY_DN895_c1_g1_i14 | (TC)6 | ACTACAACCGCGAAGATGCT | 59.90 | TTCGGGACCAAAACAAACTC | 59.95 | 257 |
